# Evaluation of the different exposure parameters for the accurate diagnosis of peri-implantitis severity in digital panoramic radiography

**DOI:** 10.4317/medoral.25501

**Published:** 2022-12-24

**Authors:** Elif Sadik, Ceren Gökmenoğlu, Gökçen Altun, Cankat Kara

**Affiliations:** 1 Orcid: 0000-0003-3382-2951. Assistant Professor, Department of Oral and Maxillofacial Radiology, Faculty of Dentistry Ordu University, Ordu, Turkey; 2 Orcid: 0000-0002-3803-7189. Associate Professor, Department of Periodontology, Faculty of Dentistry, Ordu University, Ordu, Turkey; 3 Orcid: 0000-0003-4311-6508. Assistant Professor, Department of Information Systems and Technologies, Faculty of Science, Bartın University, Bartın, Turkey; 4 Orcid: 0000-0001-5356-3665. Professor, Department of Periodontology, Faculty of Dentistry, Ordu University, Ordu, Turkey

## Abstract

**Background:**

To evaluate the accuracy of the diagnosis of peri-implant bone defects’ severities in digital panoramic radiographs obtained at different tube voltage and/or tube current settings.

**Material and Methods:**

Two different sizes of peri-implant bone defects (type 1 and type 2) were prepared after the implants were inserted into 29 bovine rib blocks. Digital panoramic radiographs were obtained at eight different tube voltage and/or tube current settings for all rib blocks. Implant images were cropped separately. The average intensity value (AIV) of cropped images were analyzed using Adobe Photoshop CC software. The Kruskal-Wallis H test was used to compare AIVs. All cropped images were evaluated using a five-point Likert scale for the likelihood of a bone defect being absent or present. The weighted kappa values were calculated to compare observer agreement and ROC analysis was performed to determine the appropriate exposure parameters.

**Results:**

The lowest AIV was obtained at 72 kV/6.3 mA (92.162±16.016), and the highest AIV was obtained at 60 kV/3.2 mA (179.050±13.823). The Kruskal-Wallis H test showed significant differences in the AIVs according to the exposure parameters (*p*<0.001). The kappa coefficient for the inter-observer agreement was excellent (0.864, *p*<0.001). The AUC values for type 1 defects ranged from 0.778 and 0.860; for type 2 defects ranged from 0.920 and 0.967. The AUC value of type 1 defects was slightly better in panoramic images obtained with high kV and low mA levels (72 kV/3.2 mA), compared to others.

**Conclusions:**

In daily clinical routine, peri-implant bone defects might be evaluated by panoramic radiographs obtained with all kV and mA values tested. However, to avoid misdiagnosing and for better accuracy, panoramic radiographs obtained with high kV and low mA levels suiTable for patients should be used, especially in the detection of small or initial bone defects.

** Key words:**Dental implant, panoramic radiography, peri-implantitis, diagnosis.

## Introduction

Osseointegrated dental implants are used to treat partially or fully edentulous patients. Osseointegration has been defined as a functional and structural connection between the newly formed bone and the implant surface. In 1-2% of patients, primary implant failure occurs due to insufficient osseointegration, within the first few months. Secondary implant failure occurs several years after successful osseointegration in about 5% of patients and is generally caused by peri-implantitis (1). Peri-implantitis is an inflammatory lesion of the tissues surrounding the osseointegrated implant, resulting in loss of supporting bone (2,3). An advanced peri-implant lesion is easily diagnosed on a radiograph by detecting the bone loss around the implant. However, in advanced cases, the prognosis of the dental implant is usually uncertain, and removal is often a better option. Peri-implant disease should be diagnosed and intervened before a substantial portion of the supporting bone is lost. If not diagnosed at the beginning and properly managed, peri-implant diseases may lead to complete loss of osseointegration and loss of the implant (2).

On radiographic images, it is difficult to identify a thin connective tissue layer lining the surface of an implant. Bone quality and density usually vary, and the contrast between implant radiopacity and adjacent bone radiolucency differs. As the cross-sections of all implants are mainly circular, the area for identification of a fibrous capsule is limited and only represents a few percent of the total surface of the implant. While diagnosing a radiolucent zone bordering a metal implant, the influence of the mach band effect is prevalent (4). Because of mach band effect, peri-implant radiolucency may occasionally be noted even in cases of successful osseointegration (5).

In terms of dental radiographic techniques, panoramic radiography (PAN) provides a view of the maxilla and mandible, but its use is limited in the radiographic evaluation of implants because of its low resolution, image distortion, and lack of cross-section information. Despite these limitations, PAN is still used because of its accessibility, ease of perform, and patient acceptance (6).

In radiological imaging, adequate image quality should be obtained while minimizing radiation exposure to patients. An increase in tube voltage and a reduction in tube current could reduce the radiation dose required but could also result in lower image quality (7). In previous studies, the effects of changes in tube voltage and tube current on image quality in digital panoramic radiographs were examined (8-10).

The aim of this *in vitro* study was to evaluate the accuracy of the diagnosis of peri-implant bone defects’ severities in digital panoramic radiographs obtained at different tube voltage and/or tube current settings. We hypothesize that a possible peri-implant defect may be overlooked, or it may be misdiagnosed by increasing the mach band effect, on radiographs obtained at kV and mA levels different from the manufacturer’s instructions.

## Material and Methods

The research protocol was approved by the Ethical Committee for Animal Research of Ordu University with the assignment protocol 2016/14. The sample size was estimated by the MedCalcversion 9.4.2.0 statistical software (MedCalc Software, Mariakerke, Belgium), yielding an alpha value of 0.05 and for the test power of 90% the sample size of 29 was set for each group.

- Bovine Rib and Implant Cavity Preparation

Fresh bovine ribs free of soft tissues were cut into 58 blocks measuring approximately 45x15x13 mm (length x height x thickness) to simulate the posterior region of the human mandible (6,11). The distal region of the ribs with a smaller diameter corresponds to D2 to D3 bone density in the Misch classification (12), while the more proximal region with a larger diameter corresponds to D3 to D4 density (13). Therefore, only the distal region of the ribs were included in this study. The cortical area, superior border of 29 rib blocks randomly selected from the cut ribs, was considered as alveolar bone, and removed with round burs until the cancellous bone became apparent. The front and back cortical plates were left intact. The purpose of this procedure was to place the implants to be localized directly into the cancellous bone, which would entirely surround the bone defects. Implants were inserted into bone blocks by the same periodontist. Three implant cavities were created in each bone sample at approximately 8 mm intervals according to the manufacturer's recommendations. One of the randomly selected implant cavities was prepared without peri-implant bone defect as the control. The other two implant cavities were prepared with peri-implant bone defect in random order, one was prepared with a ø4.1 mm countersink drill to generate 0.40 mm peri-implant bone defect (Type 1), and the other one was prepared with a ø4.8 mm countersink drill to generate 0.75 mm peri-implant bone defect (Type 2). If any bone crack occurred during the drilling process, these rib blocks were excluded from the study and replaced by others. To imitate a clinical situation for the bone defects, 40% formic acid was applied to the widened neck region of the implant cavities in the type1 and type 2 groups, with a cotton swab for about 30 seconds, and then rinsed with tap water (14). Formic acid was not applied to the control group.

All the ribs were frozen between each procedure to minimize moisture loss. Rib blocks with implant cavity and unprocessed rib blocks were grouped as pairs, where each pair was stabilized by an individual dental stone base and simulated the spatial positioning and perimeter of the mandible body. Totally 29 mandible models were made. In each model, a placement guide was made on the anterior border of the rib blocks with four artificial mandibular anterior teeth, providing uniform positioning of the implants and for appropriate positioning of rib blocks during panoramic exposure. Bone-level implants with a width of 3.3 mm and a length of 10 mm were placed on each of the implant cavities. No healing caps or abutments were placed.

- Exposures

The ribs were covered by a 16 mm layer of pink wax before scanning, for simulating the soft tissue covering of the alveolar bone and attenuation of the beam at a level equivalent to soft tissue (15). Digital panoramic images of the specimens were obtained using the Kodak 8000C Digital Panoramic and Cephalometric System (Carestream Health, Rochester, NY) which is a charge-coupled device-based system. Operating tube voltage varies from 60 kilovoltage (kV) to 90 kV and operating tube current varies from 2 milliampere (mA) to 15 mA.

The occlusal planes of the specimens were horizontally placed on the chin rest of the panoramic machine and the laser orientation beams were used to align the rib accurately in a reproducible position. Digital panoramic images of the randomly selected specimen were obtained at tube voltage increasing by 10% intervals, tube current increasing by 25% intervals, starting from the minimum exposure parameters of 60 kV/2.0 mA, in all combinations. Obtained images were examined by two periodontists and one oral and maxillofacial radiologist. AccepTable image quality was judged by consensus and proper exposure values were determined. Eight panoramic images were taken of each specimen at 60kV/3.2 mA, 66kV/2 mA, 66 kV/3.2 mA, 60kV/6.3 mA, 72kV/3.2 mA, 66 kV/6.3 mA, 66 kV/8 mA, 72 kV/6.3 mA. All images of each specimen were obtained by the same operator without changing their positions.

- Image Evaluation

**Figure 1:**
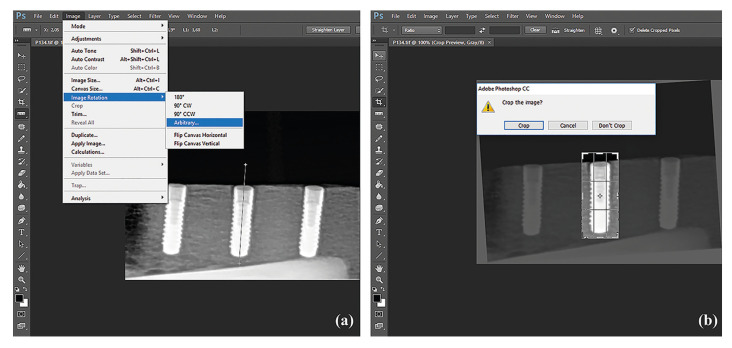
Processing the panoramic radiographs to be evaluated. (a) The long axis of the implant was defined, and the image was rotated. (b) A rectangular bounding box was drawn, and images were cropped separately.

**Figure 2:**
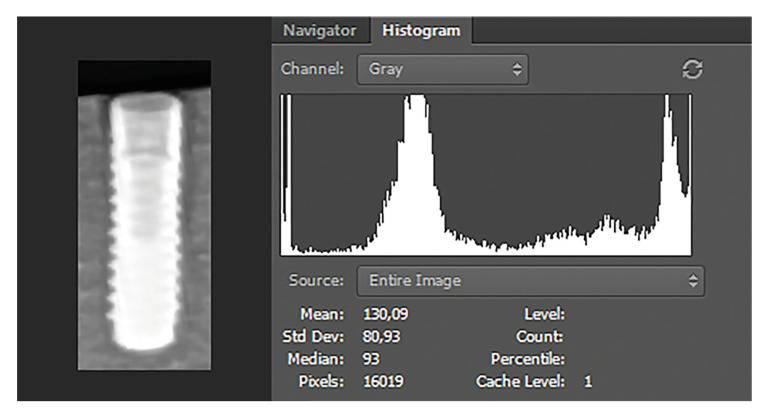
Histogram used for average intensity value measurements.

The images were exported as tagged image file format (TIFF) from Kodak software. The panoramic radiographs were processed using Adobe Photoshop CC software (Adobe Photoshop; Adobe Systems Incorporated, San Jose, CA, USA), as follows: (i) Image » Mode » Grayscale » 8 bits/ Channel was selected from the menu, (ii) A line was drawn on the long axis of the implant, (iii) The image was rotated to orient the long axis vertically, (iv) A rectangular bounding box was drawn with the sides 1.5 mm from the mesial, distal, and superior borders of the implant and 1 mm from the inferior border. For all implant images, the dimensions of the bounding box were 6.3 x 14.6 mm (83 x 193 pixels). Images were cropped separately, and the region of interest (ROI) was created (Fig. 1). The “histogram” tool of Adobe Photoshop CC software was used to show the distribution of grayscale values in ROIs. On the histogram panel, the mean represents the average intensity value (AIV) (Fig. 2). Each shade of gray assumes a value ranging from 0 to 255, with 0 corresponding to black (the most radiolucent area), and 255 to white (the most radiopaque area) (16).

**Figure 3:**
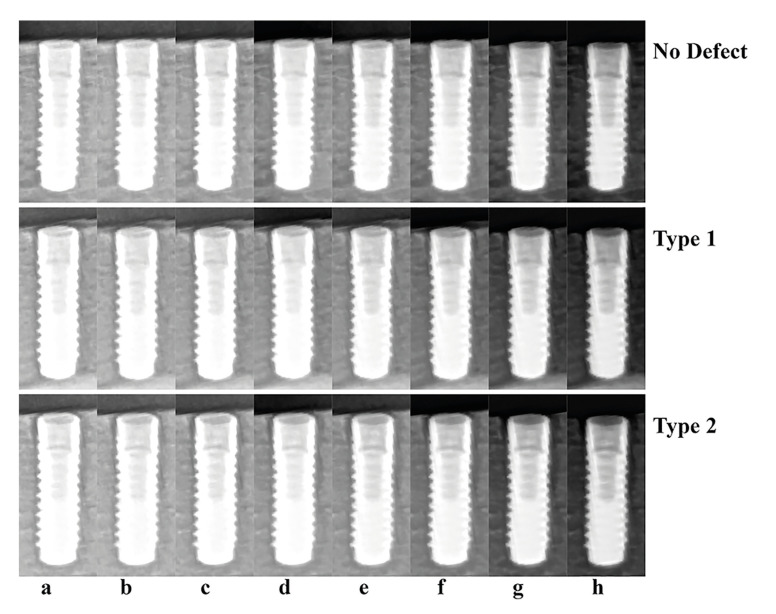
Cropped panoramic images of three different implants in the same bone block obtained with different kV and mA values; (a) 60 kV/3.2 mA; (b) 66 kV/2 mA; (c) 66 kV/3.2 mA; (d) 60 kV/6.3 mA; (e) 72 kV/3.2 mA; (f) 66 kV/6.3 mA; (g) 66 kV/8 mA; (h) 72 kV/6.3 mA.

The images were evaluated separately by three observers, each of whom had 10 to 16 years of experience in implantology or imaging applications. The ROIs (Fig. 3) were presented in random order on a 15.6-inch liquid crystal display monitor screen (EliteBook 8570p, Hewlett-Packard, Palo Alto, CA, USA), with a resolution of 1366×768 pixels. The observers had no opportunity to compare different stages of the same preparation. There was no time restriction for the evaluation.

Using image enhancement or changing the contrast and brightness adjustments was not allowed, except for image magnification. The observers scored the images on a five-point Likert scale asking whether a peri-implant bone defect was “1” definitely absent, “2” probably absent, “3” unsure, “4” probably present, “5” definitely present. Post-processing was not allowed. For control of the intra- and inter-observer differences, each observer assessed 150 images a second time after 30 days interval.

- Statistical Analysis

Statistical analysis was carried out with MedCalc version 9.4.2.0 statistical software. The Kruskal-Wallis H test was used to compare AIVs with exposure parameters and defect types. Receiver operating characteristic (ROC) analysis was performed to determine which exposure parameter was more successful in identifying defects. The weighted kappa values were calculated to measure intra- and inter-observer variations. Kendall's W coefficient was calculated for the concordance between the three observers. These results were interpreted according to the criteria of Landis and Koch (17) as 0.81 to 1 excellent agreement, 0.61 to 0.80 substantial agreement, 0.41 to 0.60 moderate agreement, 0.21 to 0.40 fair agreement, and below 0.20 poor agreement. Statistical significance was established at a *p* value less than 0.05.

## Results

**Figure 4:**
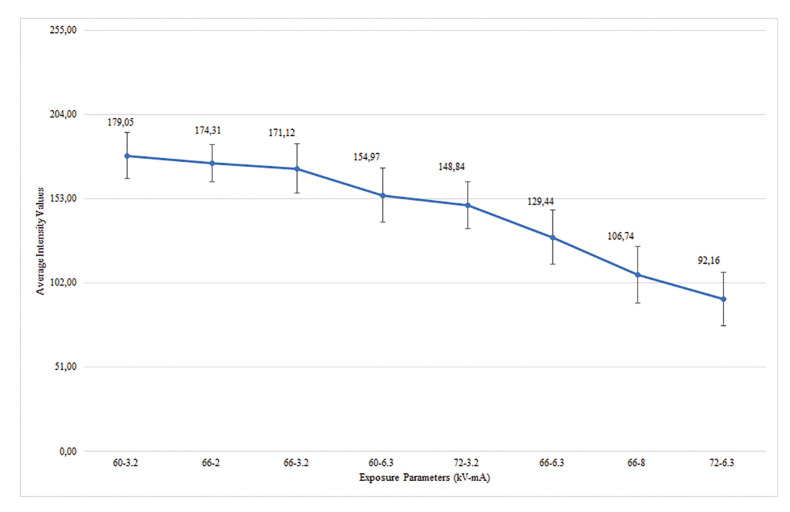
Average intensity values according to exposure parameters.

AIVs and standard deviations of the ROIs obtained with different kV and mA settings were recorded and shown in Fig. 4. While the image with the lowest AIV was obtained at 72 kV/6.3 mA setting (92.162±16.016), the image with the highest AIV was obtained at 60kV/3.2 mA setting (179.050±13.823). As shown in Table 1, the Kruskal-Wallis H test showed significant differences in the AIVs according to the exposure parameters (*p*<0.001), but there were no statistically significant differences between the AIVs and defect types (*p*>0.05). Weighted kappa statistics, standard errors, and the agreement results between the three observers examined by Kendall’s W statistics are given in Table 2. The weighted kappa coefficients for intra-observer concordance ranged between 0.778 and 0.916, which were classified as substantial and excellent agreements. Similarly, the agreement between observers 1-2 and 1-3 (0.826 and 0.841, respectively) were excellent, while between observers 2-3 (0.730) was substantial. An excellent agreement was found among the three observers (0.864, *p*<0.001).

The areas under the curve (AUC) from the ROC curves are shown in Table 3 for each exposure parameter and each type of peri-implant defect. The AUC values for type 1 defects ranged from 0.778 and 0.860; for type 2 defects ranged from 0.920 and 0.967.

**Table 1:**
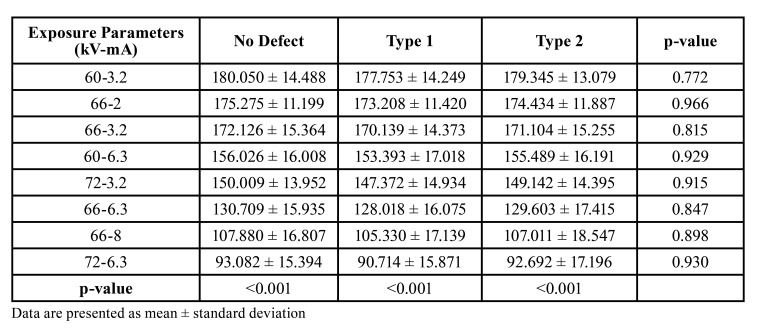
Comparison of average intensity values according to exposure parameters and defect types.

**Table 2:**
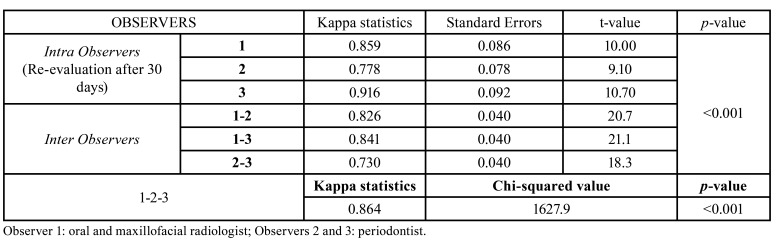
The consistency of the scores given by three different observers in the evaluation of peri-implant bone defects.

**Table 3:**
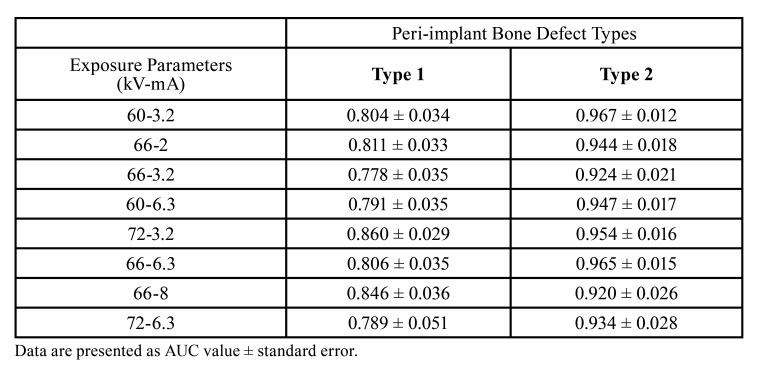
Diagnostic accuracy for detectability of peri-implant bone defects obtained at different exposure parameters: mean areas under ROC curve.

Although the AUC values for both type 1 and type 2 defects were generally high, the AUC values for the type 2 defect were higher than for the type 1 defect. The AUC value of type 1 defects was slightly better in panoramic images obtained with high kV and low mA levels (72 kV/3.2 mA), compared to others.

## Discussion

In this *in vitro* study, we evaluated possible differences in the detection of two different sizes of peri-implant defects in digital panoramic radiographs obtained at different tube voltage and/or tube current settings. The radiographic examination provides important information for determining the amount of marginal bone loss around dental implants (2). In the literature, various radiographic methods, such as intraoral radiography (IR), PAN, computed tomography (CT), and cone-beam computed tomography (CBCT), have been studied to assess the condition of the bone around dental implants. However, there is no consensus about which technique is the most accurate for evaluating bone surrounding dental implants. Kullman *et al*.(18) reported that PAN is as reliable as IR to assess bone levels around dental implants. However, in a study where peri-implant defects were evaluated with four different imaging methods (IR, PAN, CT, and CBCT) panoramic images showed greater mean deviations (0.41±0.35 mm) (19). Another study where bone loss around dental implants was evaluated using four different imaging methods, have reported the sensitivity and specificity for IR as 0.74 and 0.51, and for PAN as 0.63 and 0.43, respectively. IR with the highest sensitivity and specificity has been recommended as a favorable method for evaluating bone loss around dental implants (20). Sirin *et al*.(6) evaluated five different imaging methods for detecting different sized bone defects around dental implants. They demonstrated that AUC values were ranging from 0.72 to 0.82 for PAN, according to the type of defect. The diagnostic precision of CBCT was lower than that of conventional IR and direct digital radiography, but higher than that of multislice CT and PAN. They reported that PAN and multislice CT become more reliable when bone defects have at least 1.5 mm larger diameter than the implant diameter. PAN is limited for evaluating bone surrounding dental implants due to two-dimensional image production, low resolution, and image distortion, but it is still widely used. Sakakura *et al*.(21) showed that the majority of the dentists prescribed panoramic radiographs alone (63.8%) or associated with other radiographic methods (28.9%) for both the preoperative implant site assessment and follow-up. Clinicians determine the appropriate radiographic method according to the clinical situation. Because PAN may be preferred for the follow-up of dental implants, we constituted our study on this technique.

In this *in vitro* study, we preferred bovine ribs because they have similar characteristics to the human mandible and are easily accessible (22). The bovine rib has been used in many studies evaluating peri-implant bone defects (23). Peri-implant bone defects, that are created with the implant drills, have well-defined borders and easily imaged outlines. But in a clinical situation, peri-implant bone defects may be more diffuse, irregular in shape, and therefore more difficult to detect (5). Therefore, after bone defect preparation, we applied 40% formic acid to the defect margins for produced defects with poorly defined borders similar to in clinical cases (6,14). Soft tissue changes the absorption of the x-ray and increases the scatter radiation, which may affect the contrast and density of the film. So various materials simulating soft tissues are used in in-vitro studies to imitate the clinical situation. Dental wax and acrylic plates are the most frequently used materials (15). In this study, we used dental wax in 16 mm thicknesses as a soft-tissue simulator. Since TIFF is a widely accepted format for gray-scale images and also corresponds to the original file format of the image without compression, we exported the digital panoramic images of specimens as TIFF from Kodak software (24).

To date, several studies were published examining the image quality in digital panoramic radiographs obtained at different exposure parameters (8-10). Dula *et al*.(8) reported that digital panoramic images can be obtained by reducing the kV level from 69 to 60 without losing the diagnostic quality of radiolucent defects. Studies evaluating the effects of mA level on image quality have reported that 25% and 50% reduction of mA level did not affect the image quality (9,10). Based on these studies evaluating the effects of reducing the radiation dose on the image quality, we evaluated the effects of changing kV and mA levels during panoramic image acquisition on the diagnosis of peri-implant defects.

First described by Ernst Mach in 1865, the mach band is a perceptual phenomenon, where contrast between a dark and a relatively lighter area which is sharply demarcated is enhanced (25,26). Due to this phenomenon, the borders of adjacent areas of different radiographic densities, such as implant and bone, appear to have larger density differences than really exist (27). For this reason, the mach band can increase the number of false-positive interpretations. Factors affecting the perception of mach band are projection, background, contour, film density, and object density (28). The perception of mach band is maximal in properly exposed radiographs, much less in underexposed or overexposed films. Additionally, the ability to perceive mach band seems to vary from one observer to another (25).

The gray tones of a radiograph can be quantified, especially in digital or digitized radiographs, with the help of software. Several software programs have been used to analyze digital radiographs, including Adobe Photoshop®, VixWin®, Image Tool®, and Digora® (16). In this study, we used Adobe Photoshop® software to analyze gray tones of digital panoramic images obtained at different exposure parameters.

The main limitation of this study is using an *in vitro* setup. But we designed an *in vitro* study to be able to use many repeated radiation exposures consecutively at different kV and mA values. However, in a clinical setting, patient-related variables such as superimposition of adjacent structures, type of implants, other metal artifacts, patient positioning, and motion artifacts may affect the diagnosis. Furthermore, in this study standardized peri-implant bone defects were used, and defects were created artificially. Another limitation is using only one panoramic machine.

## Conclusions

Within the limitations of the present *in vitro* study, it is possible to conclude that the ability of PAN to detect peri-implant defects is sufficient, especially for large peri-implant bone defects. In daily clinical routine, peri-implant bone defects might be evaluated by panoramic radiographs obtained with all kV and mA values tested. However, to avoid misdiagnosing and for better accuracy, panoramic radiographs obtained with high kV and low mA levels suiTable for patients should be used, especially in the detection of small or initial bone defects. In addition, we think that more studies are needed to evaluate the accuracy of the diagnosis of bone defects around dental implants made with different materials and shapes, in digital panoramic radiographs with different exposure parameters.
